# Boosting isoprene production via heterologous expression of the Kudzu isoprene synthase gene (*kIspS*) into *Bacillus* spp. cell factory

**DOI:** 10.1186/s13568-017-0461-7

**Published:** 2017-08-08

**Authors:** Lamis Gomaa, Michael E. Loscar, Haggag S. Zein, Nahed Abdel-Ghaffar, Abdelhadi A. Abdelhadi, Ali S. Abdelaal, Naglaa A. Abdallah

**Affiliations:** 1 Agricultural Genetic Engineering Research Institute, ARC, Giza, 12619 Egypt; 2 Chair of Chemistry of Biogenic Resources, Technical University of Munich, Schulgasse 16, 94315 Straubing, Germany; 3 Department of Genetics, Faculty of Agriculture, Cairo University, Giza, 12613 Egypt; 4 Department of Genetics, Faculty of Agriculture, Damietta University, Damietta, Egypt

**Keywords:** Isoprene, Isoprene synthase, *Bacillus subtilis*, *Bacillus licheniformis*

## Abstract

Isoprene represents a key building block for the production of valuable materials such as latex, synthetic rubber or pharmaceutical precursors and serves as basis for advanced biofuel production. To enhance the production of the volatile natural hydrocarbon isoprene, released by plants, animals and bacteria, the Kudzu isoprene synthase (*kIspS*) gene has been heterologously expressed in *Bacillus subtilis* DSM 402 and *Bacillus licheniformis* DSM 13 using the pHT01 vector. As control, the heterologous expression of *KIspS* in *E. coli* BL21 (DE3) with the pET28b vector was used. Isoprene production was analyzed using Gas Chromatography Flame Ionization Detector. The highest isoprene production was observed by recombinant *B. subtilis* harboring the pHT01-*kIspS* plasmid which produced 1434.3 μg/L (1275 µg/L/OD) isoprene. This is threefold higher than the wild type which produced 388 μg/L (370 μg/L/OD) isoprene, when both incubated at 30 °C for 48 h and induced with 0.1 mM IPTG. Additionally, recombinant *B. subtilis* produced fivefold higher than the recombinant *B. licheniformis*, which produced 437.2 μg/L (249 μg/L/OD) isoprene when incubated at 37 °C for 48 h induced with 0.1 mM IPTG. This is the first report of optimized isoprene production in *B. licheniformis*. However, recombinant *B. licheniformis* showed less isoprene production. Therefore, recombinant *B. subtilis* is considered as a versatile host for heterologous production of isoprene.

**Electronic supplementary material:**

The online version of this article (doi:10.1186/s13568-017-0461-7) contains supplementary material, which is available to authorized users.

## Introduction

Isoprene is a small volatile hydrophobic molecule containing five carbon atoms and is also known as 2-methyl-1,3-butadiene. It is a colorless organic compound that is produced by animals, plants and bacteria. It has a low solubility in water as well as a low boiling point of 34 °C which enables withdrawal from the upper gas phase of a bioreactor when produced via biotechnological processes (Xue and Ahring 2011). This aspect turns it valuable for downstream chemical products. Isoprene as biofuel contains more energy, is not miscible in water and does not show corrosive effects compared to ethanol (Atsumi and Liao 2008; Lindberg et al. 2010). Companies develop bioisoprene production such as Genencor and Goodyear, published their efforts to develop a gas-phase bioprocess for production of isoprene (Whited et al. 2010). Their work involved metabolic engineering of *E. coli* capable of producing high yields of isoprene, in addition to developing a large-scale fermentation process with high rates of the isoprene recovered from the off-gas. They reported a titer of over 60 g/L, a yield of 11% isoprene from glucose and a volumetric productivity of 2 g/L/h (Chandran et al. 2011). Several researchers reported using isoprenol as anti-knock agent, in which branched C5 alcohols store more energy than ethanol and high octane numbers (RON, or research octane number, of 92–102), that helps their use as gasoline alternatives and as anti-knock additives (Cann and Liao 2010; Mack et al. 2014). In addition, they have been verified in various engine types and they proved to have better gasoline-like properties than ethanol (Yang et al. 2010). All isoprenoids are known to be derived from the two universal five-carbon (C5) building blocks, isopentenyl diphosphate (IPP) and its isomer dimethylallyl diphosphate (DMAPP). These universal precursors are known to be produced by either one of the three identified pathways: the mevalonate (MVA) pathway and/or the pathway, which is also 1-deoxy-d-xylulose-5-phosphate (DXP) pathway. Additionally, an alternative MVA-independent pathway has been identified for the biosynthesis of IPP and DMAPP in bacteria, algae and plants which is named the methylerythritol 4-phosphate (MEP) pathway and lacks the first two steps of the DXP pathway (Rodríguez-Concepción and Boronat 2012). *Bacillus subtilis* uses the DXP pathway and was found to be the best naturally isoprene producing bacteria (Kuzma et al. 1995). It is known that the isoprene synthase utilize dimethylallyl diphosphate (DMAPP) as substrate (Withers et al. 2007). The isoprene synthase gene was not identified in bacteria yet, however it has been characterized from many plants such as Populus species, e.g. aspen, *Poplar Alba* (Beatty et al. 2014; Fortunati et al. 2008; Miller et al. 2001; Sasaki et al. 2005; Sharkey et al. 2005; Silver and Fall 1995; Chotani et al. 2013; Vickers et al. 2010), *Pueraria Montana* (kudzu) and/or *Pueraria lobata* (Beatty et al. 2014; Hayashi et al. 2015; Sharkey et al. 2005). The isoprene synthase gene from poplar was successfully isolated and heterologously expressed in *E. coli*. Moreover, isoprene synthase cDNA was isolated from *Populus alba* (PaIspS) and expressed in *E. coli* for enzymatic characterization (Sasaki et al. 2005). Previous studies failed to isolate the isoprene synthase from bacteria (Julsing et al. 2007; Sivy et al. 2002), thus the Kudzu isoprene synthase gene (*kIspS*) was codon optimized and heterologously expressed in *E. coli* (Zurbriggen et al. 2012). Previously, the codon optimized *Mucuna bracteata IspS* was engineered in *S. cerevisiae* and it only produced 16.1 μg/L (Hayashi et al. 2015). Additionally, when the codon optimized *M. bracteata IspS* was engineered in *Pantoea ananatis*, it produced 63 μg/L (Hayashi et al. 2015). Recent studies involved in overexpression of codon optimized kudzu *IspS* (*kIspS*) in *E. coli* using different constructs (Cervin et al. 2016). The *E. coli* best isoprene production yield was 10 μg/L. In addition, the codon optimized kudzu and poplar *IspS* genes were expressed in *Yarrowia lipolytica* using different methods; in which the isoprene yield was 0.5–1.0 μg/L from the headspace culture (Cervin et al. 2016). This study aimed to develop recombinant *Bacillus* strains (*B. subtilis* and *B. licheniformis*) with high level of isoprene production using the Kudzu isoprene synthase.

## Materials and methods

### Construction of recombinant pET-28b plasmid with the *kIspS* for expression in *E. coli*

**Fig. 1:**
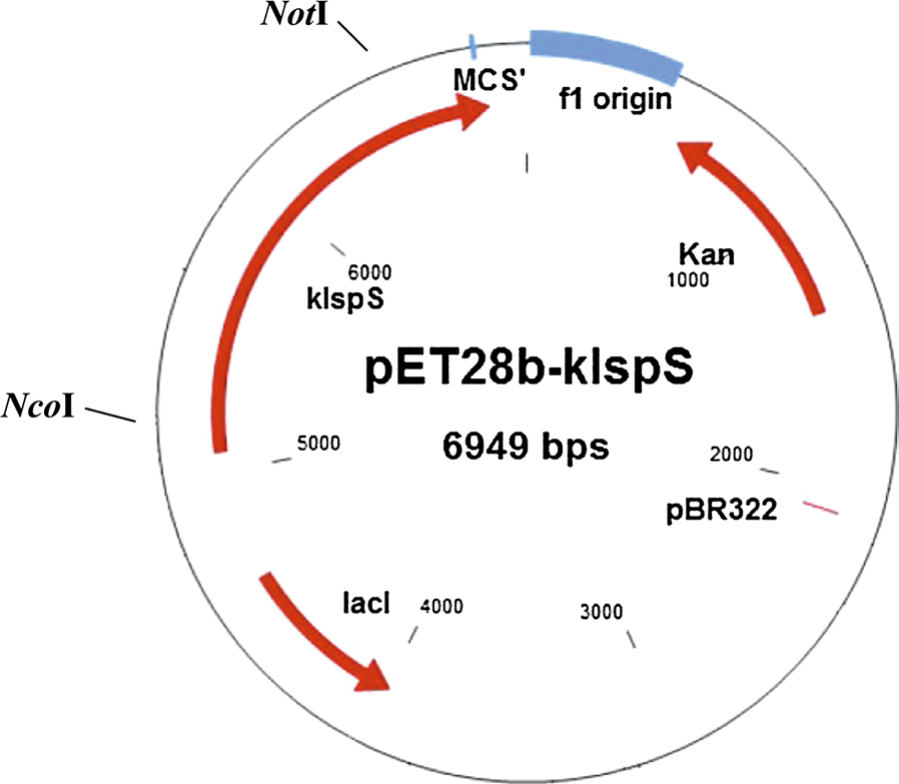
Physical map of the constructed plasmid pET28b containing the *kIspS* gene

The Kudzu isoprene synthase from pBA2kIKmA2 plasmid was kindly obtained from Anastasios Melis (Addgene plasmid #39213) (Lindberg et al. 2010). The isoprene synthase gene from *P. montana* (kudzu) presented in GenBank under Accession No. AY316691 (Sharkey et al. 2005). The *kIspS* orf (1.7 kb) was amplified using the specific primers; *kIspS*_*Nco*I_F: 5′-AACACCATGGATGCCGTGGATTT-GTGCTACGAGC-3′ and *kIspS*_*Not*I_R: 5′-ATCCGCGGCCGCCACGTACATTAGTT-GATTGATTGG-3′ with added sites *Nco*I and *Not*I, respectively to facilitate the cloning process using phusion polymerase (NEB #M0530S). The amplification reaction was performed using PCR profile with an initial cycle at 95 °C for 5 min, followed by 30 cycles 95 °C for 30 s, 58 °C for 30 s, 72 °C for 2 min and a final extension at 72 °C or 5 min. Thereafter, PCR product was cleaned up using DNA, RNA and protein purification REF 740609.250 Gel and PCR clean up protocol (January 2012/Rev.02). The purified fragment was digested with *Nco*I and *Not*I and ligated (using T4 DNA ligase NEB #002025) at the corresponding sites of pET-28b, forming the constructed plasmid pET28b-*kIspS* (Fig. 1).

### Construction of recombinant pHT01 plasmid with the *kIspS* for heterologous expression in *Bacillus*

**Fig. 2:**
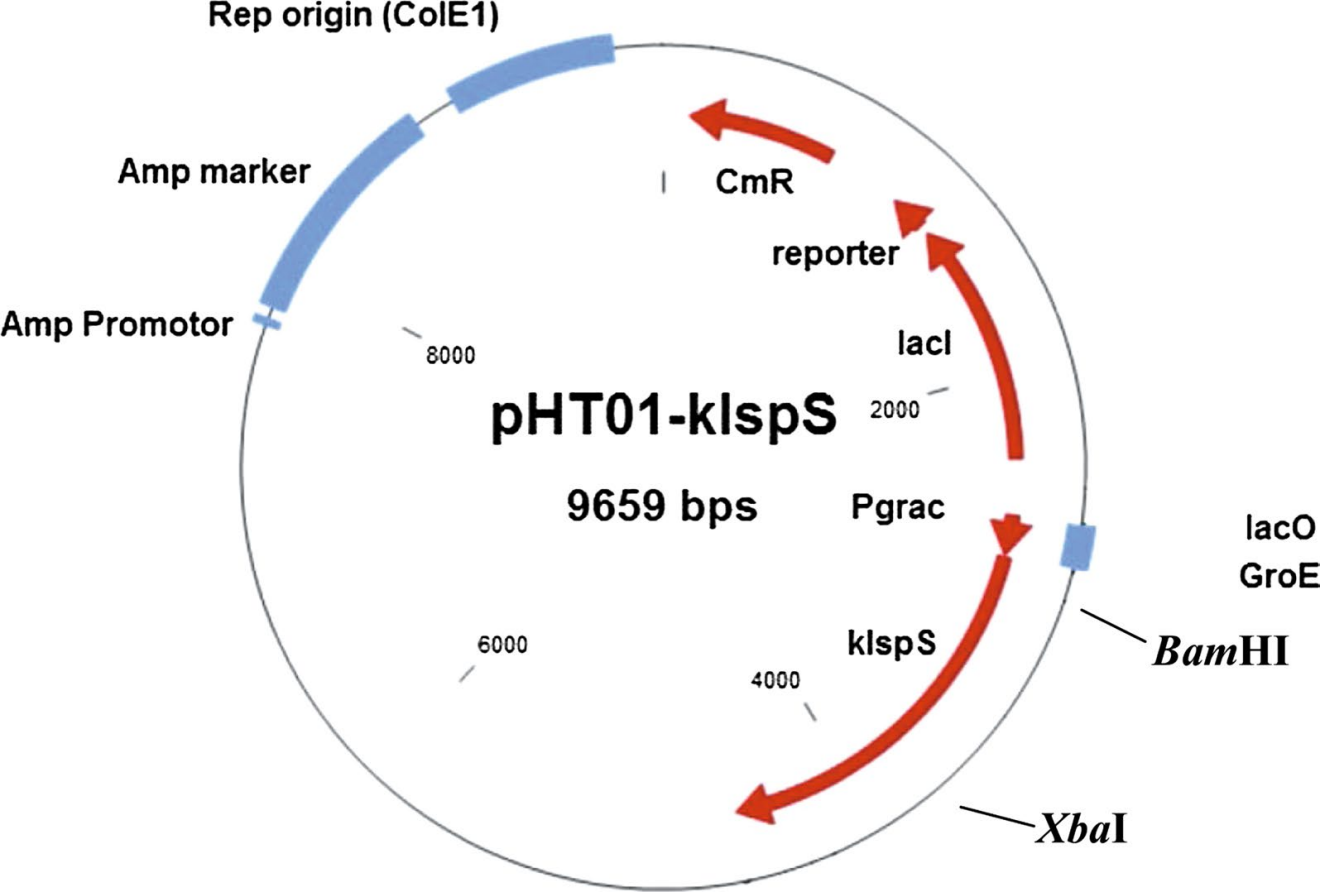
Physical map of the constructed plasmid pHT01 containing the *kIspS* gene

The pHT01 plasmid (MoBiTec GmbH), which bears chloramphenicol resistance, was used for cloning and expressing the *kIspS* gene into two *Bacillus* strains; *B. subtilis* DSM 402 (https://www.dsmz.de/catalogues/details/culture/DSM-402.html) and *Bacillus licheniformis* DSM 13 (https://www.dsmz.de/catalogues/details/culture/DSM-13.html). Conducted *B. subtilis* antibiogram showed that no natural resistance to chloramphenicol is present. To facilitate the cloning into pHT01, two primers were designed to amplify the *kIspS* gene harboring *Bam*HI and *Xba*I cloning site; named *kIspS*_*Bam*HI_F: 5′-ATATGGATCCATGCCGTGGATTTGTGCTACGAGC-3′ and *kIspS*_*Xba*I_R: 5′-ATATTCTAGACACGTACATTAGTTGATTGATTGG-3′, respectively with phusion polymerase (NEB #M0530S). The amplification reaction was performed using the same PCR profile as mentioned above and the PCR amplicon was cleaned as mentioned above. Following, the purified fragment was digested with *Bam*HI and *Xba*I and ligated (using T4 DNA ligase NEB #002025) into the corresponding restriction sites of pHT01 vector, resulting in the recombinant plasmid pHT01-*kIspS* (Fig. 2).

### Transformation and screening of the recombinant plasmid pET28b-*kIspS* in *E. coli* BL21 (DE3)

Chemical transformation was carried out for heterologous expression of pET28b-*kIspS*-C-term in BL21 (DE3) (Mamiatis et al. 1985). Colony PCR strategy was performed to screen the pET28b-*kIspS*-C-term in BL21 (DE3) using the *kIspS* specific primers with the same amplification conditions as mentioned previously.

### Transformation and screening of the recombinant plasmid pHT01-*kIspS* into *B. subtilis* and *B. licheniformis*

Preparation of electro-competent cells as well as transformation protocol was carried out according to (Xue et al. 1999) with minor modifications. In the preparation of washing and/or electroporation solution, 20% glycerol was used instead of 10% glycerol. Additionally, electroporation of the cells was performed using Micropulser electroporator BioRad catalog #165–2100 and cuvette 1 mm gap for both *Bacillus* strains. In the case of *B. subtilis*, 180 ng of plasmid DNA (pHT01-*kIspS*) was added to 60 µL of electro-competent cells using program EC1; voltage: 1.8 kV and time constant: 5.7 m/s as 1 pulse. In case of *B. licheniformis*, 290 ng of the plasmid DNA (pHT01-*kIspS*) was added to 60 µL of electro-competent cells. While for electroporation of cells mode: Ag, voltage: 2.2 kV and time constant 5.6 m/s as 1 pulse. For selection LB agar plates with 34 µg/mL chloramphenicol was used. In case of *B. subtilis*, plates were incubated on 30 °C O/N, while for *B. licheniformis* plates were incubated on 37 °C O/N. pHT01-*kIspS* plasmid mini preparation was performed (Gene JET plasmid Miniprep kit Thermo-scientific #k0503) from both *Bacillus* spp., in which lysozyme (20 mg/mL) was added as 5 mg/250 resuspension solution then samples were incubated for 1 h at 37 °C with shaking for each sample. PCR was carried out for the screening of the cells harboring the recombinant the pHT01-*kIspS* plasmid using the same primers and amplification conditions as mentioned above.

### Gas Chromatography-Flame Ionization Detector (GC-FID) analysis

Analysis of the produced isoprene was carried out using GC-FID. In this assay, an overnight grown culture was transferred to a 10 mL GC headspace vial (Macherey–Nagel, Germany) resulting in a 2 mL culture with an OD_600_ of 0.1. After incubation using an orbital shaker at 150 rpm, the GC vials were placed on the RSH Plus auto-sampler (Thermo Fisher) and injected onto a Trace 1310 gas chromatograph equipped with a flame ionization detector (FID). The column used to detect isoprene was a Bond-U-Rt column 30 m, 0.25 mm as column ID, 8 µm df from Restek GmbH (Germany). Amounts of isoprene produced were estimated by comparison with a dilution of isoprene in ethyl acetate (Sigma-Aldrich). The response of the detector was linear at a range from 1 to 4 µM of isoprene, giving the calibration Eq. (1) with an R^2^ of 1.

1 $$y = 3.113*x + 0.0003$$

Different parameters were evaluated to study the level of isoprene production, including the effect of different time intervals and IPTG induction on isoprene production.

*Bacillus subtilis* samples were incubated at 30 °C and in case of *E. coli* samples were incubated at 37 °C for 4, 8, 12, 24 and 48 h with and without 0.1 mM IPTG induction. While, *B. licheniformis* WT and recombinant *B. licheniformis* harboring pHT01-*KIspS* samples were incubated at 37 °C for 4, 8, and 48 h upon induction with 0.1 M IPTG and without induction.

### The influence of different IPTG concentrations on isoprene production

The level of isoprene production for *B. subtilis* and *B. licheniformis* harboring the recombinant plasmid pHT01-*kIspS*, in addition to *E. coli* BL21(DE3) harboring the recombinant plasmid pET28b-*kIspS* were investigated using different IPTG concentrations (i.e., 0.1, 0.5, 1 and 2 mM) for 4 h incubation at the suitable temperature for each.

### The effect of 0.3 M NaCl on *B. subtilis* and *B. licheniformis* isoprene production

Cultures of the *B. subtilis* and *B. licheniformis* were grown in Free LB and LB treated by 0.3 M NaCl with and without 0.1 mM IPTG induction for 4 h incubation at the suitable temperature for each.

### The effect of utilizing an extra carbon source on *B. subtilis* and *B. licheniformis* isoprene production

Cultures of *B. subtilis* and *B. licheniformis* were grown in Free LB and LB containing 5 g/L glucose and 5 g/L glycerol were investigated. Cultures were also grown for 4 h at the suitable temperature for each.

### Codon usage analysis for *B. subtilis* and *B. licheniformis*

Codon usage analysis was performed, in which the codon usage preference table for each *Bacillus* spp. (*B. subtilis* DSM and *B. licheniformis*) was determined using codon usage database (http://www.kazusa.or.jp/codon/). Then the *P. montana* (kudzu) isoprene synthase (*kIspS*) original sequence of GenBank Accession No. AY316691 was optimized using the online tool optimizer (http://genomes.urv.es/OPTIMIZER/). Comparison between the *kIspS* original sequence, the codon optimized *kIspS* sequence for *B. subtilis* and *B. licheniformis* showed that there are differences in the preferred codon and that could explain the differences in the level of *kIspS* in the two species.

## Results

### Generation of recombinant plasmid pET-28b-*kIspS* for heterologous expression into *E. coli*

The pET-28b plasmid and the amplified *kIspS* fragment were digested using *Nco*I and *Not*I enzymes. Agarose gel electrophoresis for the digested DNA showed the two linearized DNA strands, one at 5.2 kb which represent the expected band of the linearized pET-28 backbone and another band at 1.7 kb, which represent the *kIspS* gene (Additional file 1: Figure S1). The two bands were cut from the gel, cleaned up and ligated, together forming the recombinant plasmid pET28b-*kIspS* for heterologous expression into *E. coli* BL21 (DE3). Bacterial cells with and without the recombinant plasmid were used to measure isoprene production. For screening of the recombinant plasmid, colony PCR was carried out using isoprene specific primers. Positive clones showed a band at 1.7 kb (Additional file 2: Figure S2).

### Generation of recombinant pHT01-*kIspS* plasmid for heterologous expression into *B. subtilis* and *B. licheniformis*

The pHT01 plasmid and the amplified *kIspS* gene were digested using *Bam*HI and *Xba*I restriction enzymes. Agarose gel electrophoresis of the digested DNA showed bands at 7.9 and 1.7 kb, which represent the linearized pHT01 plasmid and the *kIspS* gene, respectively (Additional file 3: Figure S3). The two fragments were cut from the gel, cleaned up and ligated together to form the recombinant plasmid pHT01-*kIspS*. Transformation was carried out for *B. subtilis* and *B. licheniformis* transformation with the recombinant plasmid pHT01-*kIspS*. For screening of recombinant cells, plasmid was purified and subjected to PCR analysis using the specific primers, which were mentioned above. PCR amplification of the pHT01-*kIspS* plasmid displayed a band at 1.7 kb; which represents the expected size of the *kIspS* amplicon (Additional file 4: Figure S4).

### Isoprene production by different bacterial strains

**Fig. 3:**
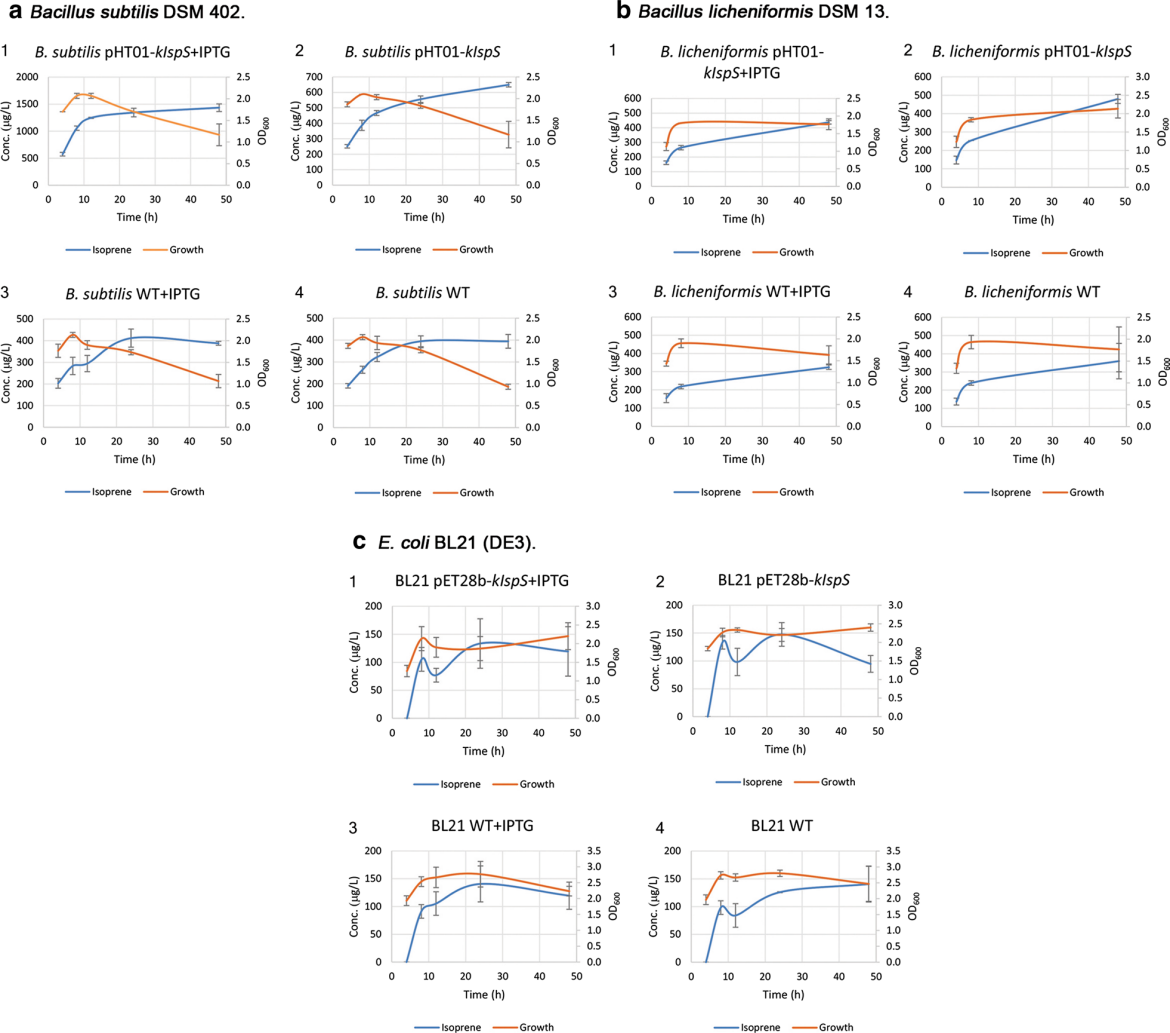
Isoprene production by different strains analyzed using Gas Chromatography Flame Ionization Detector (GC-FID). Three strains were analyzed; **a** *Bacillus subtilis* DSM 402, **b** *Bacillus licheniformis* 13 and **c** *E. coli* BL21 (DE3). Four different treatments were used; (1) recombinant strain with 0.1 mM IPTG induction, (2) recombinant strain without IPTG induction, (3) WT with 0.1 mM IPTG induction, (4) WT without IPTG induction. The average concentrations (µg/L/OD_600_) are obtained from three independent cultures starting with the standard OD_600_ nm of 0.1

The concentrations of isoprene produced from different strains were measured using GC-FID and the results indicated that the recombinant *B. subtilis* harboring pHT01-*kIspS* has the highest isoprene production of 1434.3 μg/L (1275 µg/L/OD isoprene). This is threefold higher than the wild type which produced 388 μg/L (370 μg/L/OD isoprene), when both incubated at 30 °C for 48 h and induced with 0.1 mM IPTG (Fig. 3a). While recombinant *B. licheniformis,* showed less isoprene production. Compared to the control (recombinant BL21 cells harboring pET28b-*kIspS*), which produced 53 µg/L/OD isoprene when incubated at 37 °C for 48 h and induced by 0.1 mM IPTG. Results revealed that recombinant *B. licheniformis* harboring pHT01-*kIspS* showed no significant differences in the isoprene production compared to the *B. licheniformis* WT during different time intervals (4, 8 and 48 h) of incubation at 37 °C and induction by 0.1 mM IPTG. Recombinant *B. licheniformis* harboring pHT01-*kIspS* produced 249 µg/L/OD when incubated at 37 °C for 48 h (Fig. 3b). The comparison between the expression of isoprene in the recombinant bacteria at 48 h incubation and 0.1 mM IPTG induction indicated that the two *B. subtilis* strains harboring pHT0-*kIspS* produced fivefold higher isoprene production than the recombinant *B. licheniformis* harboring pHT0-*kIspS*. To the best of our knowledge this is the first attempt of enhancing isoprene production in *B. licheniformis*.

### Influence of different IPTG concentrations on isoprene production

**Fig. 4:**
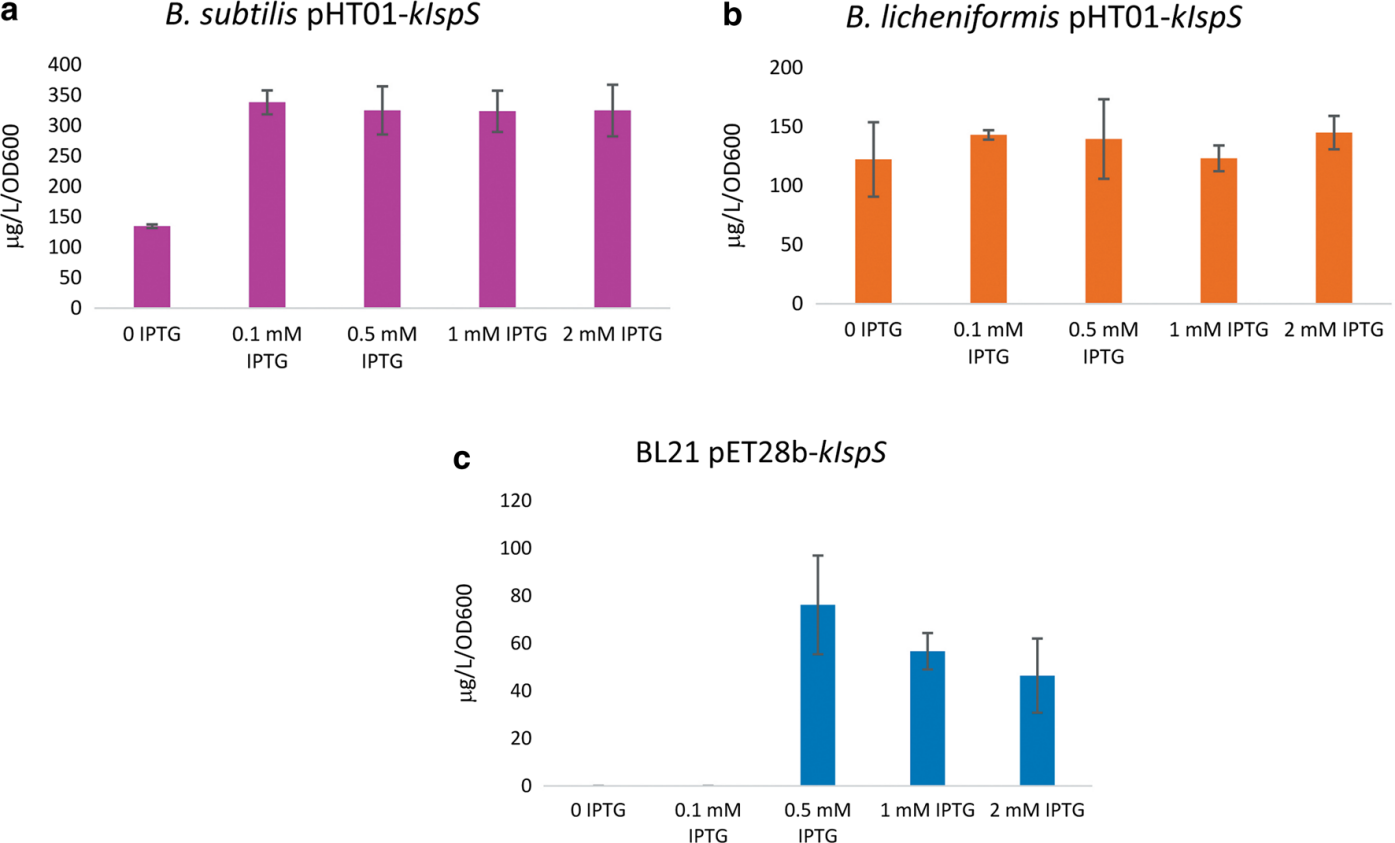
Influence of IPTG concentration on isoprene production of the different strains used in this study. The average concentrations (µg/L/OD_600_) were obtained from three independent cultures starting with the standard OD_600_ nm of 0.1. *Error bars* indicate standard deviation between replicate data

The concentrations of isoprene produced from different strains under induction with 0.1, 0.5, 1 and 2 mM IPTG were measured using GC-FID. Results showed insignificant difference in isoprene production of the recombinant *B. subtilis* harboring pHT01-*kIspS* upon different IPTG induction at 30 °C incubation for 4 h (Fig. 4a). While in case of recombinant *B. licheniformis* harboring pHT01-*kIspS*, results also showed insignificant differences in isoprene production by induction using different IPTG concentrations at 37 °C incubation for 4 h (Fig. 4b). Although recombinant *E. coli* BL21 (DE3) harboring pET28b-*kIspS* showed highest isoprene production (76 µg/L/OD) at 37 °C for 4 h incubation when induced by 0.5 mM IPTG, no isoprene production was detected for the recombinant BL21 grown in LB media induced by 0.1 mM IPTG at 37 °C incubation for 4 h (Fig. 4c).

### The effect of 0.3 M NaCl on isoprene production

**Fig. 5:**
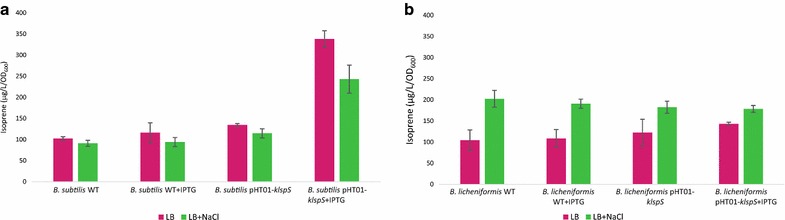
The enhancement of isoprene production in different *Bacillus* species **a** The effect of salt (0.3 M NaCl) on *Bacillus subtilis* isoprene production with and without 0.1 mM IPTG induction in WT and pHT01-kIspS recombinant *Bacillus subtilis*. **b** The effect of salt (0.3 M NaCl) on *Bacillus licheniformis* isoprene production with and without 0.1 mM IPTG induction in WT and pHT01-kIspS recombinant *Bacillus licheniformis* strain. The average isoprene production concentrations (μg per l culture per OD_600_) are obtained from three independent cultures starting with standard OD_600_ nm 0.1. The *error bars* represent the standard deviation

Our results demonstrated that 0.3 M NaCl did not enhance the isoprene production for *B. subtilis* WT, recombinant *B. subtilis* and *B. licheniformis* harboring pHT01-*kIspS*. However, 0.3 M NaCl enhanced *B. licheniformis* WT isoprene production. In this respect (Xue and Ahring 2011) reported that some external factors, such as heat 48 °C, 0.3 M NaCl and H_2_O_2_ (0.005%) induce isoprene production, while 1% ethanol inhibits isoprene production. Results revealed that *B. subtilis* harboring pHT01-*klspS* produced 243 µg/L/OD, while *B. subtilis* WT produced only 94 µg/L/OD isoprene, when both treated with 0.3 M NaCl and 0.1 mM IPTG for 4 h incubation at 30 °C. Thus, 0.3 M NaCl did not enhance the isoprene production for the recombinant *B. subtilis* harboring pHT01-*klspS*, in which it produced 338 µg/L/OD for 4 h incubation at 30 °C without NaCl and with 0.1 mM IPTG induction (Fig. 5a). While for *B. licheniformis* the WT isoprene production was slightly enhanced by 0.3 M NaCl and 0.1 mM IPTG induction, as it produced 191 µg/L/OD, compared to the *B. licheniformis* WT that produced 108.5 µg/L/OD without 0.3 M NaCl and by 0.1 mM IPTG induction at 37 °C incubation for 4 h. Recombinant *B. licheniformis* harboring pHT01-*klspS* without 0.3 M NaCl and by 0.1 mM IPTG induction, it produced 143 µg/L/OD, while when induced by 0.3 M NaCl and 0.1 mM IPTG it produced 178.5 µg/L/OD (Fig. 5b).

### Influence of utilizing an extra carbon source on isoprene production by *B. subtilis* and *B. licheniformis*

**Fig. 6:**
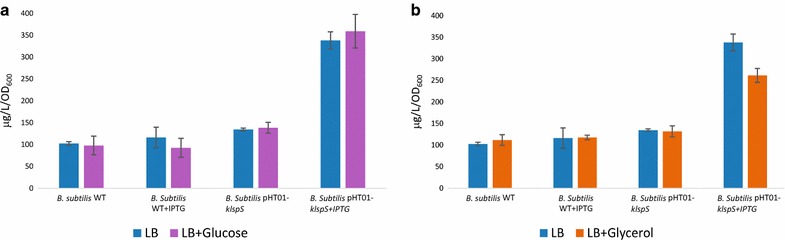
Analysis the effect of utilizing glucose and glycerol on *Bacillus subtilis* of isoprene production. **a** The influence utilizing glucose on *Bacillus subtilis* isoprene production with and without 0.1 mM IPTG induction in WT and pHT01-kIspS recombinant *Bacillus subtilis*. **b** The influence of utilizing glycerol on *Bacillus subtilis* isoprene production with and without 0.1 mM IPTG induction in WT and pHT01-kIspS recombinant *Bacillus subtilis*. The average concentrations (μg per l culture per OD_600_) are obtained from three independent cultures starting with the standard OD_600_ nm of 0.1. The *error bars* represent the standard deviation

**Fig. 7:**
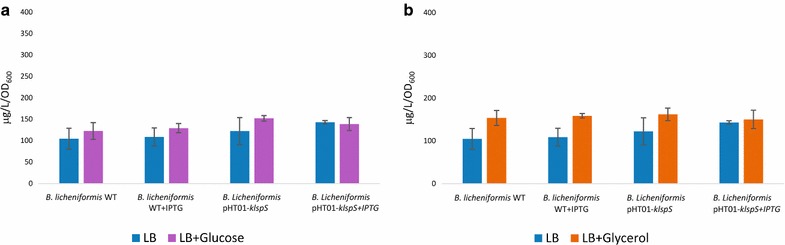
Analysis the effect of utilizing glucose and glycerol on *Bacillus licheniformis* of isoprene production. **a** The influence utilizing glucose on *Bacillus licheniformis* isoprene production with and without 0.1 mM IPTG induction in WT and pHT01-kIspS recombinant *Bacillus licheniformis*. **b** The influence of utilizing glycerol on *Bacillus licheniformis* isoprene production with and without 0.1 mM IPTG induction in WT and pHT01-kIspS recombinant *Bacillus licheniformis*. The average concentrations (μg per l culture per OD_600_) are obtained from three independent cultures starting with the standard OD_600_ nm of 0.1. The *error bars* represent the standard deviation

The highest isoprene production was observed for the recombinant *B. subtilis* harboring pHT01-*kIspS* upon utilizing 5 g/L glucose as an extra carbon source, in which it produced 359 µg/L/OD isoprene (Fig. 6a). While upon utilizing 5 g/L glycerol it produced 261.4 µg/L/OD isoprene when incubated at 30 °C for 4 h and induced by 0.1 mM IPTG (Fig. 6b). In contrary results revealed that *B. licheniformis* WT and recombinant *B. licheniformis* harboring pHT01-*kIspS* showed insignificant difference in isoprene production upon utilizing glucose (Fig. 7a) and glycerol (Fig. 7b) as an extra carbon source.

### Codon usage analysis

To clarify the reason for the difference in isoprene production in *B. licheniformis* and *B. subtilis*, bioinformatics study was carried out to predict the best codon usage for both strains using software for Codon Usage DataBase. Comparison between the *kIspS* and the optimized *IspS* sequence for *B. subtilis* and *B. licheniformis* were performed and results showed differences in the codon usage between them as shown in Additional file 5: Table S1 as shaded sequences. These differences may be the cause in the level of expression of the same gene in the different species.

## Discussion

In this study, we developed recombinant *Bacillus* strains (*B. subtilis* DSM 402 and *B. licheniformis* DSM 13) in an attempt to enhance isoprene production using the kudzu isoprene synthase. Interestingly, *B. subtilis* harboring the pHT01-*kIspS* plasmid showed a higher production of isoprene than *B. licheniformis* harboring the same plasmid. Recombinant *B. subtilis* produced 1434.3 μg/L (1275 μg/L/OD isoprene), which is threefold higher than the wild type that produced 388 μg/L (370 μg/L/OD) isoprene, when both incubated at 30 °C for 48 h by 0.1 mM IPTG induction. Our results are in accordance with a recent study for expression of *kIspS* in *B. subtilis*, in which the isoprene production levels were increased also with threefold in comparison to the wild type, from 400 μg/L to 1.2 mg/L in batch culture (Vickers and Sabri 2015). To the best of our knowledge no previous work was done for enhancing isoprene production in *B. licheniformis,* where this is the first report of optimized isoprene production in *B. licheniformis*. Since recombinant *B. subtilis* harboring pHT01-*kIspS* produce a fivefold higher isoprene production than recombinant *B. licheniformis* harboring pHT01-*kIspS* at 48 h incubation with induction by 0.1 mM IPTG. Thus, *B. licheniformis* seems to be not of significant importance for further studies on isoprene production. Additionally, multiple sequence alignment results showed differences in the codon usage of *kIspS* optimization for *B. subtilis* and *B. licheniformis*, which might be the reason that there is difference in isoprene production for both recombinant bacteria. For isoprene production optimization in our study, on one hand we found that induction by different IPTG concentrations (0.1, 0.5, 1 and 2 mM) did not change the level of isoprene production in *B. subtilis* and *B. licheniformis*. Recombinant *E. coli* BL21 (DE3) harboring pET28b-*kIspS* showed higher isoprene production (76 µg/L/OD) at 37 °C for 4 h incubation when induced by 0.5 mM IPTG. On the other hand for the effect of NaCl on isoprene production, our results demonstrated that 0.3 M NaCl did not enhance the isoprene production for all strains under study except the wild type *B. licheniformis*. However, it was revealed that NaCl and heat can induce isoprene production, (Xue and Ahring 2011), in which isoprene increases at temperature ranging between 25 and 45 °C then decreases until it reaches 0 at 65 °C in another study, optimum bacterial isoprene production was obtained at 45 °C (Kuzma et al. 1995). Moreover, when utilizing extra carbon sources to the media, i.e. glucose and/or glycerol, highest isoprene production was observed for the recombinant *B. subtilis* at 5 g/L glucose as an extra substrate. This result is in contrary to the previous observation (Zurbriggen et al. 2012) for *E. coli* transformed with *kIspS*, in which glycerol provided higher yields of isoprene compared to glucose, fructose, xylose, or LB media. In addition, isoprene production assays demonstrated that *kIspS* expression in *E. coli* best activity were obtained at 37 °C for 6 h when induced by 0.1 mM IPTG (Zurbriggen et al. 2012). Previous studies demonstrated that *Synechocystis* PCC6803 and *E. coli* are responsive strains for heterologous transformation by the *IspS* gene, in which they express and store the isoprene protein into their cytosol (Lindberg et al. 2010). Recent studies involved in overexpression of codon optimized kudzu *IspS* (*kIspS*) in *E. coli* using different constructs (Cervin et al. 2016). In This study, the *E. coli* best isoprene production yield was 10 μg/L. Moreover, the codon optimized kudzu and poplar *IspS* genes were expressed in *Y. lipolytica* using different methods; in which the isoprene yield was 0.5–1.0 μg/L from the headspace culture (Cervin et al. 2016). Previously, the codon optimized *M. bracteata IspS* was engineered in *Corynebacterium glutamicum* and produced 24.2 μg/L, additionally it was also engineered in *Enterobacter aerogenes* and produced 316 μg/L (Hayashi et al. 2015). Moreover, the *kIspS* was expressed in *Trichoderma reesei*; in which it yields 0.5 μg/L isoprene. Also, the synthetic tagged *kIspS* gene was introduced into *Synechocystis* sp. PC6803 (Lindberg et al. 2010). Results revealed that low levels of IspS protein were detected, in addition to the codon optimization that significantly enhanced protein production, in which latter strain produced 50 μg isoprene per gram dry cell weight per day, which is equivalent to 4 μg isoprene/L culture/h^−1^ (Hong et al. 2012). The same *kIspS* construct was used with a glucose-sensitive version of *Synechocystis* sp. PC6803; which produced isoprene that peaked at 100–130 μg/L culture (Bentley et al. 2014; Bentley and Melis 2012). Additionally, the isoprene production was slightly improved to 300 μg/L culture by introducing a heterologous MVA pathway (Bentley et al. 2014). There are previous studies on expression of more than one isoprene synthase, which shows are highly production of isoprene. In which the expression of ten isoprene synthase genes from Arachishypogaea together with the MVA pathway in *E. coli* resulted in the production of up to 35 mg/L/h/OD of isoprene (Beatty et al. 2014). Also, it was shown that heterologous expression of *P. alba* IspS and *S. cerevisiae* MVA pathway in *E. coli*, yield 532 mg/L isoprene in a fed-batch fermentation (Yang et al. 2012). Previous study on enhancing isoprene production through heterologous expression of *B. subtilis* DXS and DXR yield 314 mg/L isoprene, while over expression endogenous DXS and DXR in *E. coli* harboring *P. nigra* IspS gene enhanced isoprene production from 94 to 160 mg/L (Zhao et al. 2011). Additionally, by introducing RBS and nucleotide spacers provide the maximum isoprene expression in *E. coli* batch cultures from 0.4 mg/L isoprene of the control culture to 5 mg/L isoprene per of MEP super-operon transformants culture and up to 320 mg/L isoprene of MVA super-operon transformants culture (Zurbriggen et al. 2012). It was demonstrated that *B. subtilis* bears an isoprene synthase activity which utilizes the dimethylallyl diphosphate (DMAPP) as a substrate for isoprene production. Additionally, the isoprene synthase activity was optimal at pH 6.2 as well as it requires low levels of divalent ions and it was found to be separated from the chloroplast isoprene synthase (Sivy et al. 2002). Recently, 1-deoxy-d-xylulose-5-phosphate synthase (Dxs) and 1-deoxy-d-xylulose-5-phosphate reductoisomerase (Dxr) were overexpressed separately in *B. subtilis* DSM 10 strain. Over expression of Dxs increased the yield of isoprene by 40%. While over expression of Dxr had no change on the level of isoprene production (Xue and Ahring 2011). Concerning the control we have successful results in transformation of recombinant plasmid pET28b-*kIspS*-C-term in BL21 cells and the highest isoprene production for the recombinant BL21 cells harboring pET28b-*kIspS*-C-term was 70 μg/L/OD when incubated at 37 °C for 24 h induced by 0.1 mM IPTG. Previous assays for *kIspS* expression in *E. coli*, revealed that the best isoprene production activity were obtained at 37 °C for 6 h with 0.1 mM IPTG induction (Zurbriggen et al. 2012). Moreover, heterologous expression of the codon optimized *kIspS* in *E. coli* has been carried out and results showed that there is no significant difference of *kIspS* gene expression in recombinant and non-recombinant *E. coli* (Zurbriggen et al. 2012).

It can be concluded from the obtained results that recombinant *B. subtilis* is a better host than *B. licheniformis* and *E. coli* for expressing isoprene and is considered as a versatile host for heterologous production of isoprene. It is recommended in the future research to give more attention for synthetic biology as well as substrate utilization pathways that would aid enzyme optimization and production improvement.

## Additional files

**Additional file 1: Figure S1.** Digestion of pET28b plasmid and the amplified *kIspS* fragment with *Nco*I and *Not*I to 2 generate pET28b-*kIspS-*C-term construct. Marker: 2 log DNA ladder (1.0–10.0 kb) NEB catalogue #3 N3200, Lane 1: pET28b digested by *Nco*I and *Not*I (5.2 kb), Lane 2: *kIspS* (1.7 kb) digested by *Nco*I 4 and *Not*I.

**Additional file 2: Figure S2.** Colony PCR results of the recombinant plasmid pET28b-*kIspS*-C term in BL21 (DE3) cells. 7 Marker: 2 log DNA ladder (1.0–10.0 kb) NEB catalogue #N3200, Lane 1, 2 & 3 are positive results for 8 the colony PCR of pET28b-*kIspS*-C terminal in BL21 (DE3) cells.

**Additional file 3: Figure S3.** Digestion of pHT01 plasmid and the amplified *kIspS* fragment with *Bam*HI and *Xba*I to 11 generate pHT01-*kIspS* construct. Marker: 2 log DNA ladder (1.0–10.0 kb) NEB catalogue #N04695, 12 Lane 1: pHT01 (7.9 kb) digested by *Bam*HI and *Xba*I, Lane 2: amplified *kIspS* fragment (1.7 kb) 13 digested by *Bam*HI and *Xba*I.

**Additional file 4: Figure S4.** PCR screening results for recombinant *B. subtilis* and *B. licheniformis* harboring the 15 pHT01-*kIspS* plasmid. Lane 1: Negative control. Lane 2: PCR results for pHT01-*kIspS* in *B.* 16 *licheniformis*. Lane 3: positive control from pHT01-*kIspS*. Lanes 4, 5 & 6: PCR results for pHT01-17 *kIspS* in *B. subtilis.* Marker: 2 log DNA ladder (1.0–10.0 kb) NEB catalogue #N04695.

**Additional file 5: Table S1.** Differences between the *kIsps* codon and the optimized codon for (A) *B. subtilis* and (B) *B.* 20 *licheniformis*. Shadows show the differences in the codon.

## Abbreviations

*kIspS*: Kudzu isoprene synthase
GC-FID: Gas Chromatography Flame Ionization Detector
DXP: 1-deoxy-d-xylulose-5-phosphate
DMAPP: dimethylallyl diphosphate
IPP: isopentenyl diphosphate
