# Supplementary figures and images for: Boosting isoprene production via heterologous expression of the Kudzu isoprene synthase gene (kIspS) into Bacillus spp. cell factory
Source: AMB Express. 2017 Aug 8;7:161. doi: 10.1186/s13568-017-0461-7 (PMC5548705; doi:10.1186/s13568-017-0461-7)

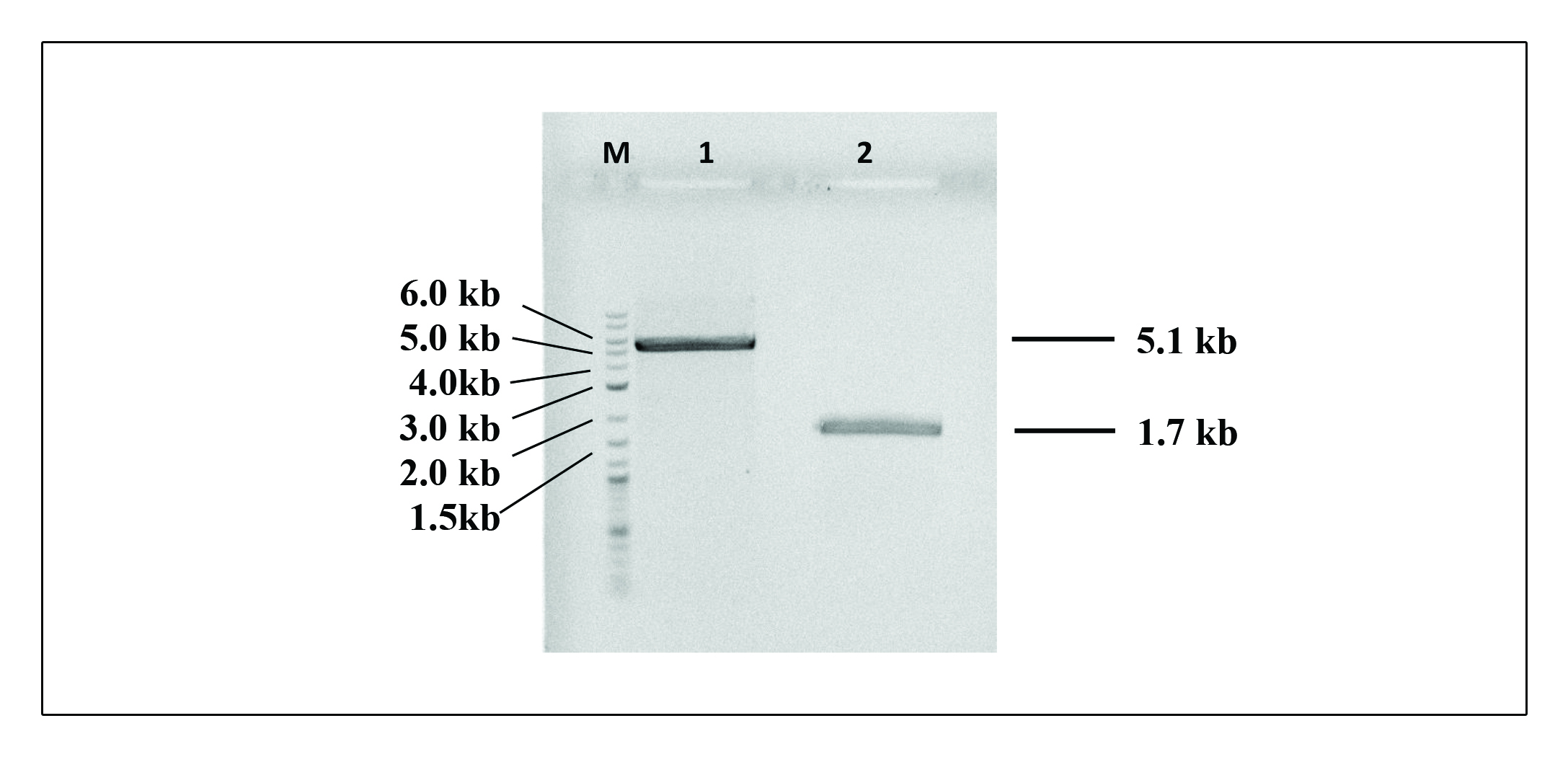

Supplement: Supplementary file 1 — Additional file 1: Figure S1. Digestion of pET28b plasmid and the amplified kIspS fragment with NcoI and NotI to 2 generate pET28b-kIspS-C-term construct. Marker: 2 log DNA ladder (1.0–10.0 kb) NEB catalogue #3 N3200, Lane 1: pET28b digested by NcoI and NotI (5.2 kb), Lane 2: kIspS (1.7 kb) digested by NcoI 4 and NotI. [file 13568_2017_461_MOESM1_ESM.jpg]

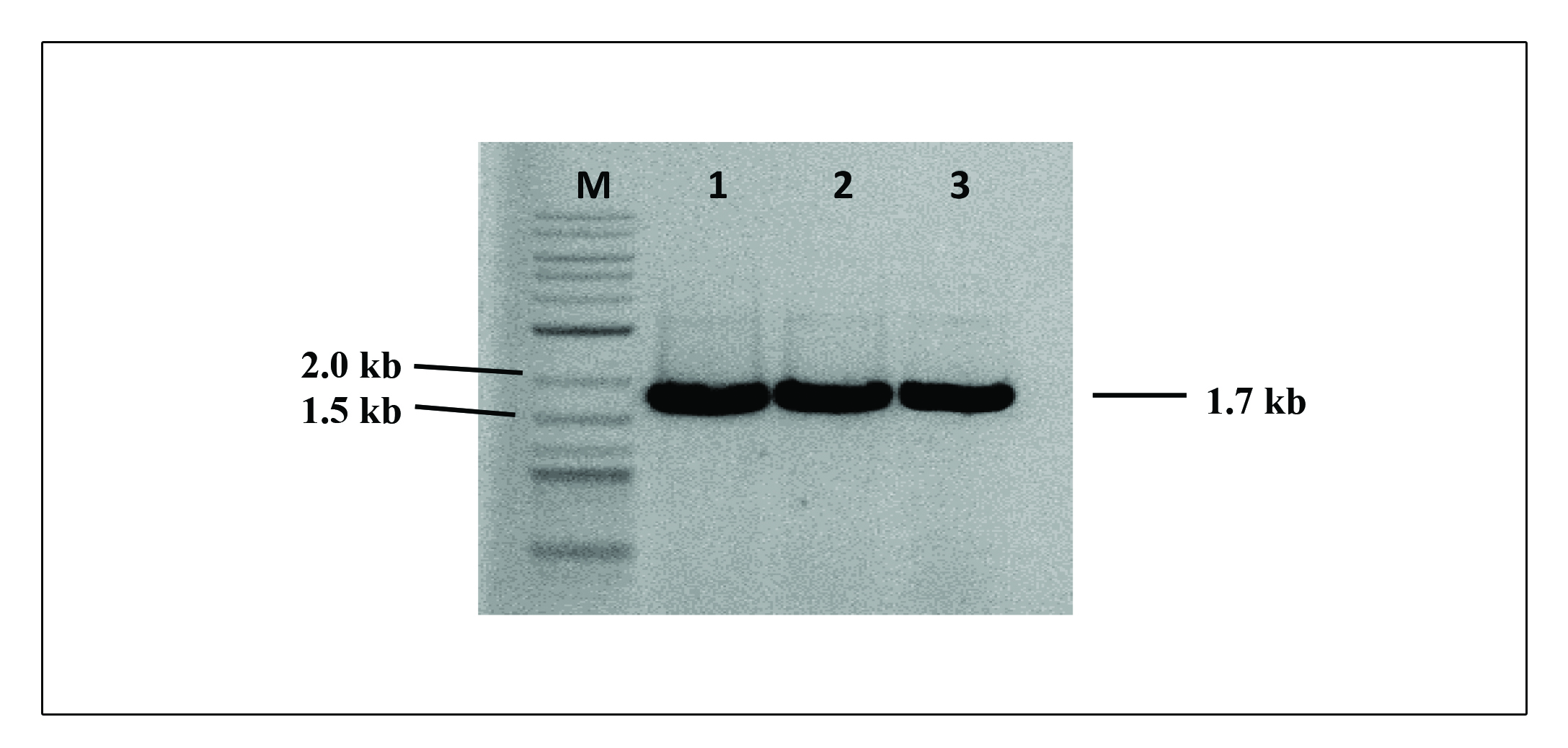

Supplement: Supplementary file 2 — Additional file 2: Figure S2. Colony PCR results of the recombinant plasmid pET28b-kIspS-C term in BL21 (DE3) cells. 7 Marker: 2 log DNA ladder (1.0–10.0 kb) NEB catalogue #N3200, Lane 1, 2 & 3 are positive results for 8 the colony PCR of pET28b-kIspS-C terminal in BL21 (DE3) cells. [file 13568_2017_461_MOESM2_ESM.jpg]

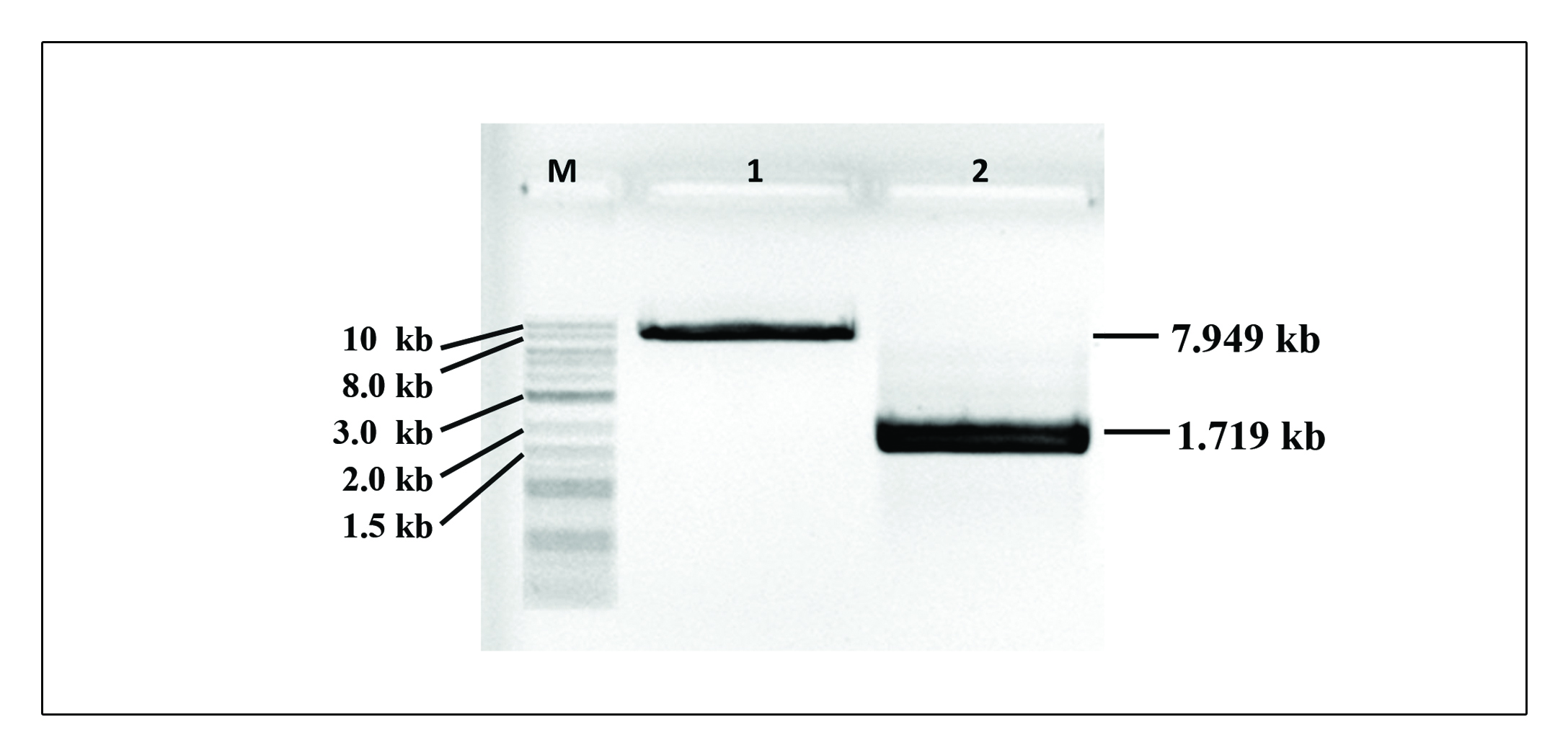

Supplement: Supplementary file 3 — Additional file 3: Figure S3. Digestion of pHT01 plasmid and the amplified kIspS fragment with BamHI and XbaI to 11 generate pHT01-kIspS construct. Marker: 2 log DNA ladder (1.0–10.0 kb) NEB catalogue #N04695, 12 Lane 1: pHT01 (7.9 kb) digested by BamHI and XbaI, Lane 2: amplified kIspS fragment (1.7 kb) 13 digested by BamHI and XbaI. [file 13568_2017_461_MOESM3_ESM.jpg]

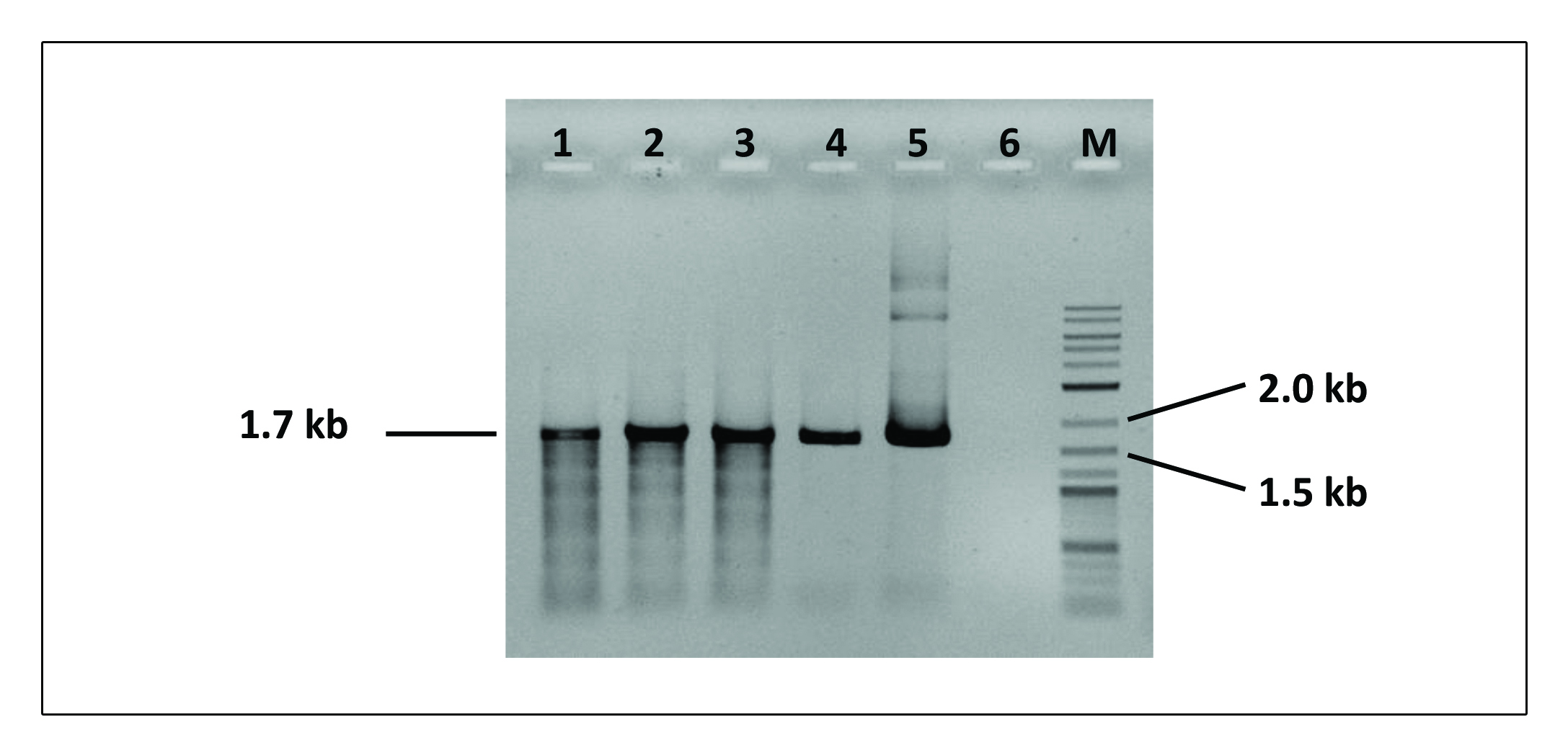

Supplement: Supplementary file 4 — Additional file 4: Figure S4. PCR screening results for recombinant B. subtilis and B. licheniformis harboring the 15 pHT01-kIspS plasmid. Lane 1: Negative control. Lane 2: PCR results for pHT01-kIspS in B. 16 licheniformis. Lane 3: positive control from pHT01-kIspS. Lanes 4, 5 & 6: PCR results for pHT01-17 kIspS in B. subtilis. Marker: 2 log DNA ladder (1.0–10.0 kb) NEB catalogue #N04695. [file 13568_2017_461_MOESM4_ESM.jpg]
