# Supplementary material for: Boosting isoprene production via heterologous expression of the Kudzu isoprene synthase gene (kIspS) into Bacillus spp. cell factory
Source: AMB Express. 2017 Aug 8;7:161. doi: 10.1186/s13568-017-0461-7 (PMC5548705; doi:10.1186/s13568-017-0461-7)
Supplement: Supplementary file 5 — Additional file 5: Table S1. Differences between the kIsps codon and the optimized codon for (A) B. subtilis and (B) B. 20 licheniformis. Shadows show the differences in the codon. [file 13568_2017_461_MOESM5_ESM.docx]

**(A) *B. subtilis***

| **Codons** | **Query** | **Optimized** | **Codons** | **Query** | **Optimized** | **Codons** | **Query** | **Optimized** | **Codons** | **Query** | **Optimized** |
| --- | --- | --- | --- | --- | --- | --- | --- | --- | --- | --- | --- |
| **GCA (A)** | 17 | 0 | **GCC (A)** | 4 | 0 | **GCG (A)** | 2 | 37 | **GCT (A)** | 14 | 0 |
| **TGC (C)** | 8 | 0 | **TGT (C)** | 2 | 10 | **GAC (D)** | 19 | 0 | **GAT (D)** | 15 | 34 |
| **GAA (E)** | 24 | 52 | **GAG (E)** | 28 | 0 | **TTC (F)** | 12 | 28 | **TTT (F)** | 16 | 0 |
| **GGA (G)** | 10 | 0 | **GGC (G)** | 4 | 23 | **GGG (G)** | 2 | 0 | **GGT (G)** | 7 | 0 |
| **CAC (H)** | 3 | 0 | **CAT (H)** | 12 | 15 | **ATA (I)** | 9 | 0 | **ATC (I)** | 12 | 25 |
| **ATT (I)** | 4 | 0 | **AAA (K)** | 22 | 40 | **AAG (K)** | 18 | 0 | **TTA (L)** | 9 | 0 |
| **TTG (L)** | 18 | 0 | **CTA (L)** | 17 | 0 | **CTC (L)** | 9 | 0 | **CTG (L)** | 8 | 81 |
| **CTT (L)** | 20 | 0 | **ATG (M)** | 13 | 13 | **AAC (N)** | 15 | 34 | **AAT (N)** | 19 | 0 |
| **CCA (P)** | 11 | 0 | **CCC (P)** | 5 | 0 | **CCG (P)** | 0 | 20 | **CCT (P)** | 4 | 0 |
| **CAA (Q)** | 21 | 0 | **CAG (Q)** | 5 | 26 | **AGA (R)** | 11 | 0 | **AGG (R)** | 4 | 0 |
| **CGA (R)** | 7 | 0 | **CGC (R)** | 1 | 31 | **CGG (R)** | 1 | 0 | **CGT (R)** | 7 | 0 |
| **AGC (S)** | 6 | 0 | **AGT (S)** | 7 | 0 | **TCA (S)** | 9 | 45 | **TCC (S)** | 9 | 0 |
| **TCG (S)** | 4 | 0 | **TCT (S)** | 10 | 0 | **ACA (T)** | 16 | 34 | **ACC (T)** | 7 | 0 |
| **ACG (T)** | 4 | 0 | **ACT (T)** | 7 | 0 | **GTA (V)** | 3 | 0 | **GTC (V)** | 6 | 0 |
| **GTG (V)** | 12 | 0 | **GTT (V)** | 9 | 30 | **TGG (W)** | 10 | 10 | **TAC (Y)** | 8 | 0 |
| **TAT (Y)** | 12 | 20 | **TAA (.)** | 1 | 1 | **TGA (.)** | 0 | 0 | **TAG (.)** | 0 | 0 |

**(B) *B. licheniformis***

| **Codons** | **Query** | **Optimized** | **Codons** | **Query** | **Optimized** | **Codons** | **Query** | **Optimized** | **Codons** | **Query** | **Optimized** |
| --- | --- | --- | --- | --- | --- | --- | --- | --- | --- | --- | --- |
| **GCA (A)** | 17 | 0 | **GCC (A)** | 4 | 0 | **GCG (A)** | 2 | 37 | **GCT (A)** | 14 | 0 |
| **TGC (C)** | 8 | 10 | **TGT (C)** | 2 | 0 | **GAC (D)** | 19 | 0 | **GAT (D)** | 15 | 34 |
| **GAA (E)** | 24 | 52 | **GAG (E)** | 28 | 0 | **TTC (F)** | 12 | 0 | **TTT (F)** | 16 | 28 |
| **GGA (G)** | 10 | 0 | **GGC (G)** | 4 | 23 | **GGG (G)** | 2 | 0 | **GGT (G)** | 7 | 0 |
| **CAC (H)** | 3 | 0 | **CAT (H)** | 12 | 15 | **ATA (I)** | 9 | 0 | **ATC (I)** | 12 | 25 |
| **ATT (I)** | 4 | 0 | **AAA (K)** | 22 | 40 | **AAG (K)** | 18 | 0 | **TTA (L)** | 9 | 0 |
| **TTG (L)** | 18 | 0 | **CTA (L)** | 17 | 0 | **CTC (L)** | 9 | 0 | **CTG (L)** | 8 | 81 |
| **CTT (L)** | 20 | 0 | **ATG (M)** | 13 | 13 | **AAC (N)** | 15 | 34 | **AAT (N)** | 19 | 0 |
| **CCA (P)** | 11 | 0 | **CCC (P)** | 5 | 0 | **CCG (P)** | 0 | 20 | **CCT (P)** | 4 | 0 |
| **CAA (Q)** | 21 | 26 | **CAG (Q)** | 5 | 0 | **AGA (R)** | 11 | 31 | **AGG (R)** | 4 | 0 |
| **CGA (R)** | 7 | 0 | **CGC (R)** | 1 | 0 | **CGG (R)** | 1 | 0 | **CGT (R)** | 7 | 0 |
| **AGC (S)** | 6 | 45 | **AGT (S)** | 7 | 0 | **TCA (S)** | 9 | 0 | **TCC (S)** | 9 | 0 |
| **TCG (S)** | 4 | 0 | **TCT (S)** | 10 | 0 | **ACA (T)** | 16 | 0 | **ACC (T)** | 7 | 0 |
| **ACG (T)** | 4 | 34 | **ACT (T)** | 7 | 0 | **GTA (V)** | 3 | 0 | **GTC (V)** | 6 | 30 |
| **GTG (V)** | 12 | 0 | **GTT (V)** | 9 | 0 | **TGG (W)** | 10 | 10 | **TAC (Y)** | 8 | 0 |
| **TAT (Y)** | 12 | 20 | **TAA (.)** | 1 | 1 | **TGA (.)** | 0 | 0 | **TAG (.)** | 0 | 0 |
